# Dosimetric and radiobiological comparison of simultaneous integrated boost radiotherapy for early stage right side breast cancer between three techniques: IMRT, hybrid IMRT and hybrid VMAT

**DOI:** 10.1186/s13014-022-02009-2

**Published:** 2022-03-28

**Authors:** Suyan Bi, Rui Zhu, Zhitao Dai

**Affiliations:** 1 National Cancer Center/National Clinical Research Center for Cancer/Cancer Hospital & Shenzhen Hospital, Chinese Academy of Medical Sciences and Peking Union Medical College, Shenzhen, 518116 China; 2 Department of Oncology, Yunyang County People’s Hospital, Chongqing, 404500 China

**Keywords:** Breast cancer, Second cancer risk, IMRT, Hybrid IMRT, Hybrid VMAT

## Abstract

**Purpose:**

This study aimed at evaluating the clinical impact of full intensity-modulated radiotherapy (IMRT), hybrid IMRT (H-IMRT) and hybrid volumetric-modulated arc therapy (H-VMAT) for early-stage breast cancer with simultaneous integrated boost (SIB), in terms of plan quality and second cancer risk (SCR).

**Methods:**

Three different plans were designed in full IMRT, hybrid IMRT, and hybrid VMAT for each of twenty patients with early-stage breast cancer. Target quality, organs at risk (OARs) sparing, and SCR were compared among the three plans for each case.

**Results:**

In compared with H-IMRT, IMRT plans showed deterioration in terms of *D*_2%_ of SIB, *V*_10_ of ipsilateral lung, and excess absolute risk (EAR) to contralateral lung (C-Lung) and esophagus. *D*_2%_ and the homogeneity index (HI) of SIB, V5 of ipsilateral lung (I-Lung), the *D*_*mean*_ of the esophagus, the EAR to C-Lung and the esophagus with hybrid VMAT dramatically increased by 0.63%, 10%, 17.99%, 149.27%, 230.41%, and 135.29%, respectively (*p* = 0.024; 0.025; 0.046; 0.011; 0.000; 0.014). *D*_*mean*_ of the heart, the EAR to contralateral breast (C-Breast) and C-Lung by full IMRT was significantly decreased in comparison to the H-VMAT (4.67%, *p* = 0.033, 26.76%, *p* = 0.018; 48.05%, *p* = 0.036).

**Conclusion:**

The results confirmed that H-IMRT could achieve better target quality and OARs sparing than IMRT and H-VMAT for SIB radiotherapy of early-stage right breast cancer. H-IMRT was the best treatment option, while H-VMAT performed the worst among the three plans in terms of SCR to peripheral OARs.

## Introduction

Usually diagnosed as early-stage female cancer, the 5-year specific survival rate of breast cancer is up to 98.9% [1]. Whole breast radiotherapy (RT) and a boost to the tumor bed is considered as the adjuvant therapy after breast-conserving surgery for early-stage breast cancer [2, 3]. Studies confirmed that patients benefited from RT and tumor bed boosting [3, 4].

Various RT techniques, such as three-dimensional conformal radiation therapy (3D-CRT), intensity-modulated radiation therapy (IMRT), and volumetric-modulated arc therapy (VMAT), have been adopted for treating breast cancer. Utilizing two opposed, wedged, and tangential fields, 3D-CRT treating the whole breast is carried out with multi-leaf collimators (MLCs) to shield the adjacent normal tissue. Many studies [5–7] have been confirmed that tangential field techniques such as dynamic wedge and field-in-field techniques are used for whole-breast radiation can improve dose uniformity to the tumour.

3D-CRT has the advantage of improving the local control, but the toxicities associated with radiation to the organs at risk (OARs) are a concern [8]. Dividing each beam into smaller beamlets, IMRT delivers a non-uniform fluence to optimize the dose distribution [8]. VMAT can rotate the angle of gantry and radiate beams continuously, and modulate the dose rate (DR) and the shape of the MLCs simultaneously to achieve a highly conformal dose coverage [9]. IMRT and VMAT were reported to have incomparable advantages in dose homogeneity and coverage compared with 3D-CRT [9, 10]. However, IMRT might be more susceptible to setup error and shape changes of the breast in whole breast RT [10]. To reduce the effects of the geometrical uncertainties, Nakamura et al. [11] proposed a method of hybrid IMRT plan comprised of two opposed tangential open beams and two inverse-planned IMRT beams. And they proved the hybrid IMRT had excellent performance in target quality and offsetting the geometrical uncertainties for patients who underwent whole breast RT [12].

With the advancement of medical technology, systemic therapy and radiation therapy techniques have greatly lengthened the life span of women with breast cancer. This, however, may increase the likelihood of radiation-induced secondary cancers. RT resulted in inevitably radiation damage and therapy-related second cancer risk (SCR) for normal tissue, which was confirmed by studies [12, 13]. With the improvement of the efficacy and overall survival of breast cancer patients, the SCR and radiation toxicity caused by RT has gradually become a research focus. Although IMRT, hybrid IMRT, hybrid VMAT and VMAT have been shown to improve dose conformity and reduce dose to organs at risk (OARs) compared with 3D-CRT, organ doses to out-of-field regions were greater with IMRT or VMAT than with 3D-CRT, due to the former methods having greater scattering and monitor unit (MU) [14–17]. Early studies showed that 3D-CRT possesses a lower SCR than IMRT and VMAT for the [18, 19]. In clinical breast cancer treatment, however, the uniformity of the target area and the dose of normal tissue should be considered simultaneously. When considering early stage breast cancer with SIB radiotherapy treatment, 3D-CRT technique may result in worse target uniformity compared with IMRT and VMAT techniques. Therefore, 3D-CRT was usually replaced by modern intensity modulation technology in SIB treatment for early stage breast cancer.

To pursue an excellent target dose coverage and OARs sparing, and also lower the SCR and radiation toxicity, selecting a reasonable RT modality is critical for treating breast cancer. To the best of our knowledge, the clinical impact of hybrid VMAT for early stage breast cancer with SIB treatment has not been studied. This study aimed at assess the plan quality and SCR among three treatment modalities (full IMRT, hybrid IMRT, and hybrid VMAT) for SIB treatment of early stage breast cancer.

## Materials and methods

### Patients preparation

Twenty females aged between 31 and 64 years old, with early-stage right-sided breast cancer after breast-conserving surgery, were randomly selected. None of the patients had contraindications for RT. This study was approved by the ethics committee of National Cancer Center/National Clinical Research Center for Cancer/Cancer Hospital & Shenzhen Hospital, and the informed consent was acquired from each enrolled patient.

All of the patients were positioned with a breast bracket and fixed foam plate on the affected side of the lower limbs. The computed tomography (CT) scans were acquired on a Philips Brilliance Big Bore CT (Philips, Holland) simulation in 5-mm-thick slices, in the supine position with the scan scope from the mandible to the thorax. In addition, all of the adjacent normal tissues, such as the heart, lung, esophagus, and contralateral breast, were completely covered.

### Contouring of target volumes and OARs

Target volumes and OARs were delineated on the Eclipse treatment planning system (Version 13.6, Varian Medical Systems Inc.). The clinical target volume (CTV) and the boost region were delineated by the same radiation oncologist on each CT data set. The CTV was the whole breast tissue identifiable on the CT scan assisted by wire markers, which were placed around the palpable breast tissue during the simulation. Then the CTV limited posteriorly by the intercostal front and retracted 5 mm from the skin. The boost region encompassed the surgical bed or seroma. The planning target volume (PTV) was expanded 5 mm based on the CTV, excluding the heart. Then the PTV was retracted 5 mm from the skin and limited posteriorly by the intercostal front. The boost region was expanded by 5 mm in all directions to create the SIB (simultaneous integrated boost) volume. The contoured OARs were the contralateral breast (C-Breast), heart, spinal cord, esophagus, and ipsilateral (I-Lung) and contralateral lungs (C-Lung). Before RT planning, we should also deal with the lead wire marked on the body surface during CT positioning and modify its CT values to -1000HU to reduce the impact on dose distribution. In order to avoid the target receiving insufficient radiation dose because of the target changes in size due to edema during treatment or residual displacement due to breathing not properly controlled, a 10 mm artificial expansion with soft-tissue equivalent HU was added in the breast region and the PTV contours toward the external direction.

### RT plans

**Fig. 1:**
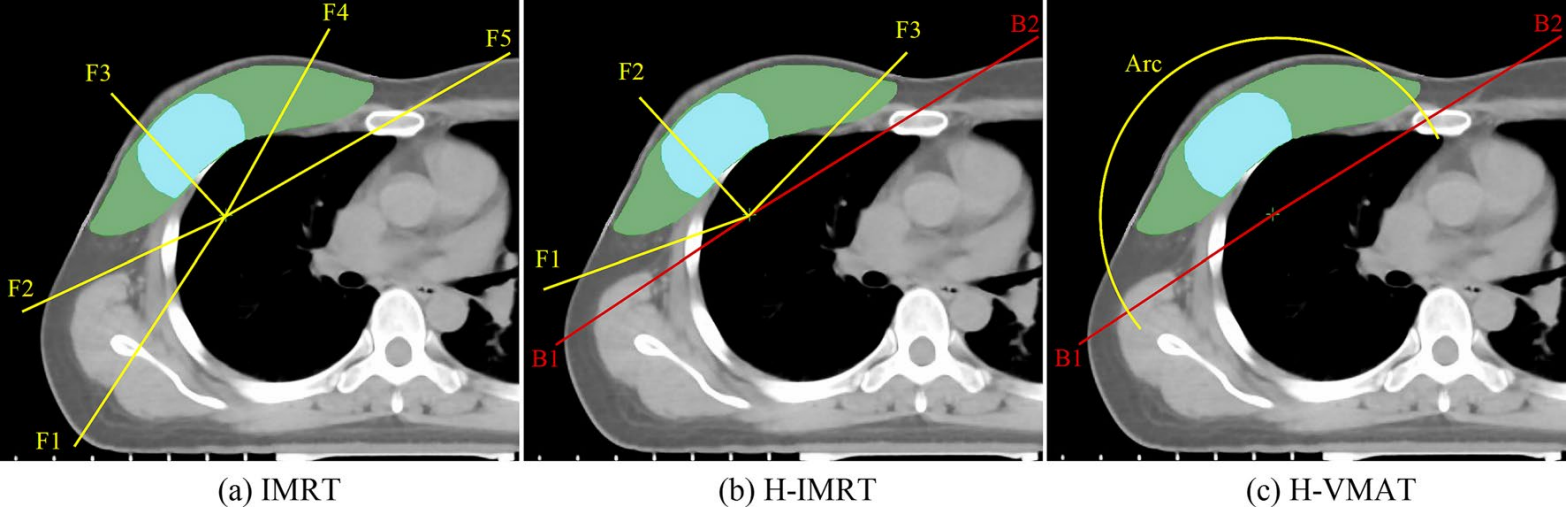
Target volume contouring and field arragement display of three irradiation techniques: **a** IMRT, **b** Hybrid IMRT (H-IMRT) and **c** Hybrid VMAT (H-VMAT). The yellow straight lines, red straight lines, and yellow arc represent IMRT beams, tangential beams and partial arc beam, respectively. The green area is the planning target volume (PTV), and the light blue area is simultaneous integrated boost (SIB)

Figure 1 showed the fields distributions in CT images for the three RT techniques respectively. Three different RT plans (full IMRT, hybrid IMRT, and hybrid VMAT) were created for each case in the Eclipse TPS. Utilizing 6 MV photon beams generated by Varian IX linear accelerator, dose optimization and calculations were done in Eclipse TPS for all of the plans. The algorithms of Dose-Volume Optimizer and Progressive Resolution Optimizer were used for IMRT, and VMAT dose optimization, respectively, and Anisotropic Analytical Algorithm was adopted for final dose calculations [20, 21]. For the purpose of comparison, all the plans were normalized so that 95% of PTV covered by 43.5 Gy. All the plans were optimized with the same dose constraints [22] as was detailed in Table 1.

**Table 1:** Dose targets and constraints for treatment planning

**S**tructure	Metrics	Objective
SIB	V_49.5Gy_ (%)	≥ 95%
	V_55Gy_ (%)	< 20%
	D_max_ (Gy)	< 60 Gy
PTV-SIB	V_43.5Gy_ (%)	≥ 95%
	V_48Gy_ (%)	< 20%
	D_max_ (Gy)	< 52 Gy
I-Lung	V_5Gy_ (%)	< 60%
	V_10Gy_ (%)	< 40%
	V_20Gy_ (%)	< 20%
	V_30Gy_ (%)	< 15%
	D_mean_ (Gy)	< 15 Gy
C-lung	V_5Gy_ (%)	< 20%
Heart	D_mean_ (Gy)	< 4 Gy
C-Breast	D_max_ (Gy)	< 40 Gy
	D_mean_ (Gy)	< 3 Gy
Spinal cord	D_max_ (Gy)	< 45 Gy

#### Full IMRT

The full IMRT plans contained two opposed tangential fields, and the other four fields that were at the angles of 10° or 20° to the two tangential fields in the direction of outside the body. The angles of the collimator and the position of jaws of all of the fields were adjusted before dose optimization to maximize the protection of the lungs. All of the fields were delivered with dynamic sliding-window IMRT delivery technique and the fixed DR of 600 monitor units (MUs)/min.

#### Hybrid IMRT

The hybrid IMRT plans owned two opposed tangential open beams plus three IMRT beams. Two of the three IMRT beams were at the angles of 10° to the two tangential fields in the direction of outside the body, and the third IMRT beam had an angle of about 30° to 45° to the tangential field on the upper side avoiding exposure to the heart and contralateral breast. To maximize the protection of the lungs, the angles of the collimator of the three IMRT beams were adjusted, and the position of the jaws of the third IMRT beam was adjusted and fixed, adapting the shape of the SIB before dose optimization and calculation. The adopted delivery technique and DR were the same as that of the full IMRT plans. The open beams contributed 80% of the total dose, whereas the inversely optimized IMRT beams contributed to the remaining prescription dose.

#### Hybrid VMAT

The hybrid VMAT plans owned two opposed tangential open beams and a half arc beam. The gantry of the arc beam rotated from one tangential angle to the other tangential angle. The maximum DR of the arc beam was set to 600 MUs/min. The open beams contributed with 80% of the total dose, whereas the inversely optimized arc beams contributed to the remaining prescribed dose.

For the SIB and PTV-SIB of all of the plans, the prescribed doses were 49.50 and 43.50 Gy in 15 fractions, respectively. The prescribed 95% isodose covered no less than 95% of the target volume [23], and the percentage volume of the target volume radiated over 110% of the prescribed dose was no more than 2%. The dose constraints for adjacent OARs of contralateral breast, heart, ipsilateral lung, contralateral lung, spinal cord, and esophagus were defined according to published literature [24]. According to the planning method of Giorgia Nicolini [25], in order to avoid the target receiving insufficient radiation dose because of the target changes in size due to edema during treatment or residual displacement due to breathing not properly controlled, we give a 10 mm artificial expansion with soft-tissue equivalent HU of the body in the breast region and of the PTV contours toward the external direction for the full IMRT and VMAT plans.

### Treatment plan evaluation

The data collected from the Dose-Volume Histogram (DVH) of all of the plans were evaluated in the aspect of target coverage and OARs sparing. SIB: the maximum dose (D_max_), the mean dose (D_mean_), and V_95%_ of SIB were assessed. The D_max_ of SIB, also named D_2%_, is defined as the dose received by 2% of the target volume, and V_95%_ is defined as the percentage volume of the target volume receiving 95% of the prescribed dose. The conformal index (CI) and homogeneity index (HI) were also evaluated. The CI of SIB is defined as $CI = TV_{PTV}^{2} /\left( {TV \times PIV} \right)$ utilizing the Paddick conformity index, where the ${\mathrm{TV}}_{\mathrm{PTV}}$ was the SIB volume receiving 95% of the prescription dose, the TV is the total volume of the SIB, and the PIV is the total volume covered by the prescribed 95% isodose. The HI of SIB was assessed using *HI* = *(D*_*5%*_*-D*_*95%*_*)*/*D*_*mean*_, where D_5%_ and D_95%_ are the minimum dose radiated to 5% and 95% of the SIB, respectively. PTV-SIB: the D_2%_, the D_mean_, V_95%,_ and CI of PTV-SIB were assessed. These indicators were defined as described above. OARs: the D_max_ and D_mean_ of contralateral breast, heart, spinal cord and esophagus, and the D_mean_ of contralateral lung were executed for dosimetric analysis. The V_5_ (the percentage volume receiving 5 Gy), V_10_ (the percentage volume receiving 10 Gy), V_20_ (the percentage of volume receiving 20 Gy), V_30_ (the percentage of volume receiving 30 Gy), and D_mean_ of the ipsilateral lung and combined lung were also evaluated.

### SCR calculations

The SCR caused by RT of normal tissues can be assessed by excess absolute risk (EAR) model, as proposed by Schneider [23, 26]. The EAR to develop a solid cancer after exposure to radiation has been estimated from data of the atomic bomb survivors for different kinds of solid cancer and describes the absolute difference in cancer rates of persons exposed to a dose d and those not exposed to a dose beyond the natural dose exposion per 10,000 person-years per Gy. The Eq. (1) shown below can be utilized to calculate the SCR of an organ [27, 28]:

1 $$EAR^{org} = \frac{1}{{V_{T} }}\sum\limits_{i} {V(D_{i} )\beta_{EAR} RED(D_{i} )\mu (x,a)}$$

where *V*_*T*_ is the total organ volume assessed for secondary carcinogenesis, *V* (*D*_*i*_) represents the organ volume receiving the dose *D*_*i*_, and the parameter *β*_*EAR*_ is the slope of the dose–response curve in the low dose region. Equation (2), RED (*D*_*i*_), represents the dose–response mechanistic model, which describes the fractionation effects and cell killing:

2 $$RED(D_{i} ) = \frac{{e^{{ - \alpha^{{\prime }} D_{i} }} }}{{\alpha^{{\prime }} R}}\left( {1 - 2R + R^{2} e^{{\alpha^{{\prime }} D_{i} }} - (1 - R)^{2} e^{{ - \frac{{\alpha^{{\prime }} R}}{1 - R}D_{i} }} } \right)$$

where *R* is a parameter that represents the repopulation or repair ability of normal tissues between two dose fractions, and the parameter *α*′ was calculated by Eq. (3):

3 $$\alpha^{{\prime }} = \alpha + \beta d = \alpha + \beta D_{i} /D_{T} d_{T}$$

where *D*_*T*_ is the prescribed dose of 49.50 Gy to the SIB in this study, and *d*_*T*_ represents the corresponding fractionation dose of 3.3 Gy. Given by Eq. (4), *µ* (*x*, *a*) expresses the modifying function:

4 $$\mu \left( {x,a} \right) = e^{[\gamma e(x - 30) + \gamma a\ln (a/70)]}$$

where *γ*_*e*_ and *γ*_*a*_ are both the age modifying parameters.

In this study, the EAR has been investigated to the organs of contralateral breast, contralateral lung, ipsilateral lung, and esophagus. The assumed value of α/β = 3 Gy for all of the organs needed to evaluate EAR, and all of the other parameters used in EAR calculation were taken from previous research [27] and were shown in Table 2.

**Table 2:** Model parameters used in EAR calculation

Structure	*β*_*EAR*_	*γ*_*e*_	*γ*_*a*_	*α*	*α/β*	*R*
C-Breast	9.2	− 0.037	1.7	0.044	3	0.15
I-Lung	7.5	0.002	4.23	0.042	3	0.83
C-Lung	7.5	0.002	4.23	0.042	3	0.83
Esophagus	0.58	− 0.002	1.9	0.026	3	0.81

### Statistical analysis

All the parameters were calculated from the DVHs. Statistical analyses were carried out using IBM SPSS Statistics version 21 (SPSS Inc.Armonk, NY). A paired t-test was performed to analyze the difference between three techniques, and a *p* value < 0.05 was considered to reveal statistical significance.

## Results

### Target volume

**Fig. 2:**
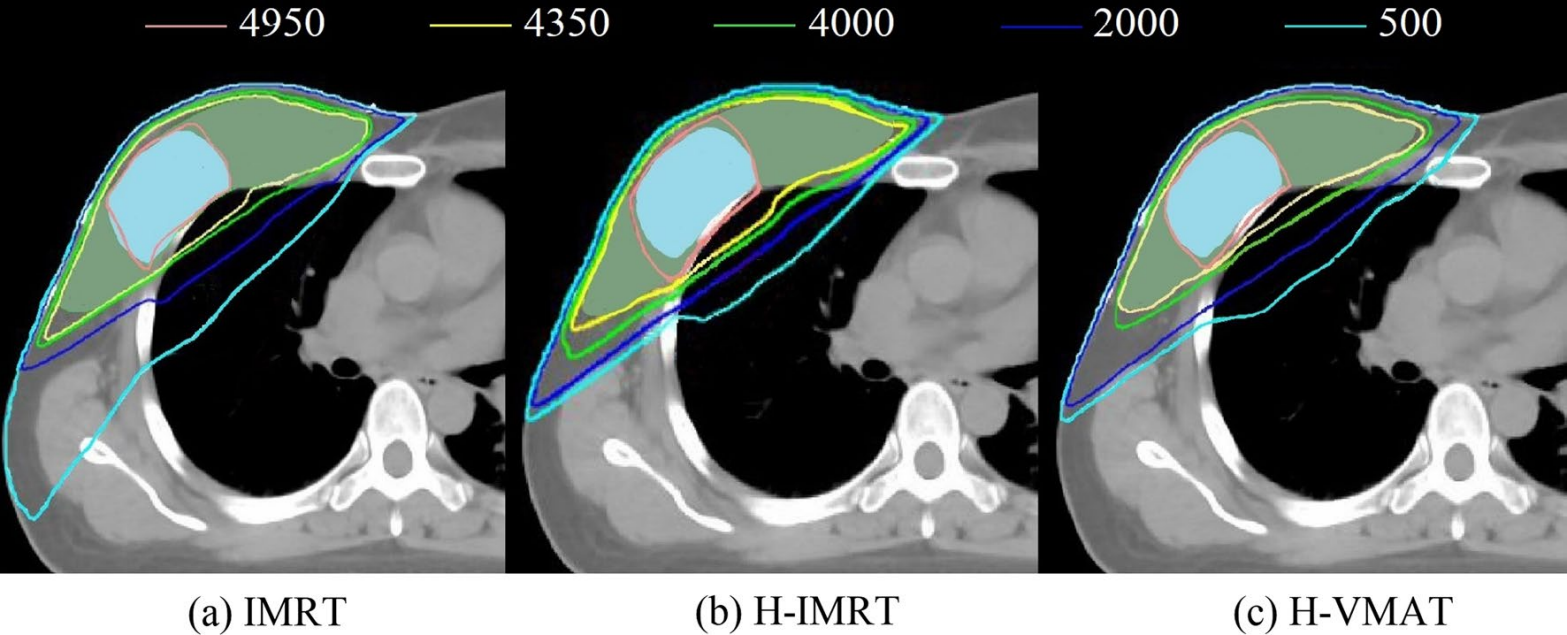
Comparison of planar dose distribution in cGy for a representative patient with three irradiation techniques: **a** IMRT, **b** Hybrid IMRT (H-IMRT) and **c** Hybrid VMAT (H-VMAT)

**Fig. 3:**
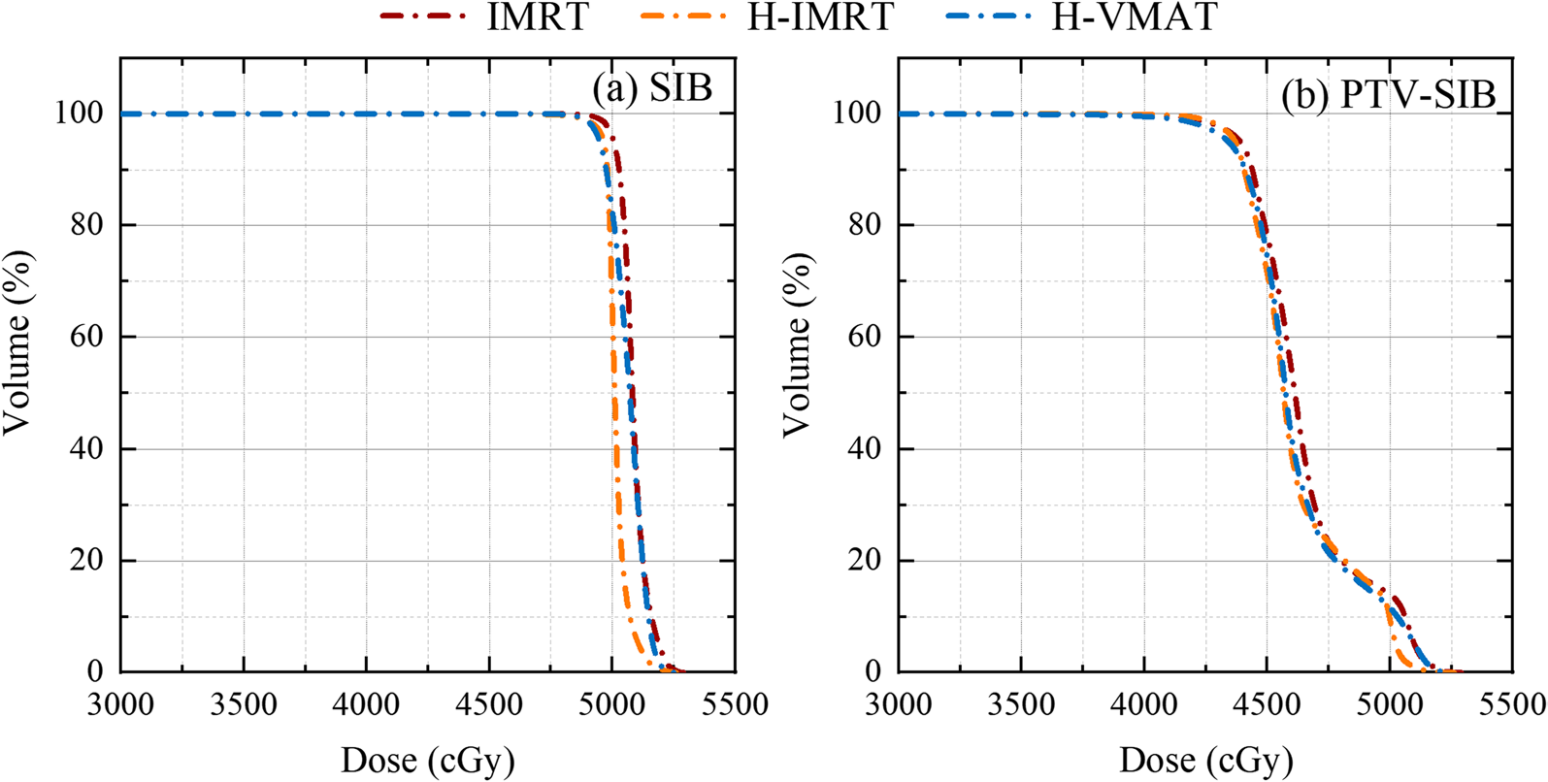
DVHs of **a** SIB and **b** PTV-SIB for the representative patient with IMRT (red line), Hybrid IMRT (orange line) and Hybrid VMAT (blue line)

The comparison of isodose lines from 500 to 4950 cGy for a selected case is illustrated in Fig. 2. The DVHs of SIB and PTV-SIB of one representative case are displayed in Fig. 3a, b, respectively. The parameters of D_2%_, D_mean_, V_95%_, CI, and HI were compared to evaluate the quality of target dose coverage. For SIB, the hybrid IMRT obtained a lower D_2%_ than both full IMRT and hybrid VMAT (*p* < 0.05) and achieved better HI than the hybrid VMAT (*p* < 0.05). For the PTV-SIB, the V_95%_ of the hybrid IMRT (99.37 ± 0.51) was better than that of the full IMRT and the hybrid VMAT (98.99 ± 0.42, 99.03 ± 0.67). The findings on SIB and PTV-SIB are listed in Table 3.

**Table 3:** Comparison of dosimetric parameters of SIB and PTV-SIB between IMRT, H-IMRT and H-VMAT

Structure	Parameters	IMRT	H-IMRT	H-VMAT	*p* value		
					a	b	c
SIB	D_2%_ (Gy)	52.73 ± 0.63	52.35 ± 0.60	52.68 ± 0.48	0.042	0.776	0.024
	D_mean_ (Gy)	51.18 ± 0.47	51.18 ± 0.59	51.41 ± 0.38	0.197	0.322	0.181
	V_95%_ (%)	99.88 ± 0.33	99.96 ± 0.11	100.00 ± 0.00	0.317	0.225	0.224
	CI	0.82 ± 0.06	0.84 ± 0.05	0.84 ± 0.04	0.202	0.163	0.747
	HI	0.12 ± 0.06	0.12 ± 0.02	0.13 ± 0.02	0.575	0.174	0.025
PTV-SIB	D_2%_ (Gy)	49.40 ± 0.68	49.29 ± 0.45	49.21 ± 0.39	0.581	0.359	0.461
	D_mean_ (Gy)	45.69 ± 0.42	45.60 ± 0.23	45.57 ± 0.28	0.438	0.264	0.660
	V_95%_ (%)	98.99 ± 0.42	99.37 ± 0.51	99.03 ± 0.67	0.06	0.621	0.076
	CI	0.63 ± 0.08	0.65 ± 0.10	0.650 ± 0.09	0.38	0.35	0.252

a: IMRT versus H-IMRT; b: IMRT versus H-VMAT; c: H- IMRT versus H-VMAT

### OARs

**Fig. 4:**
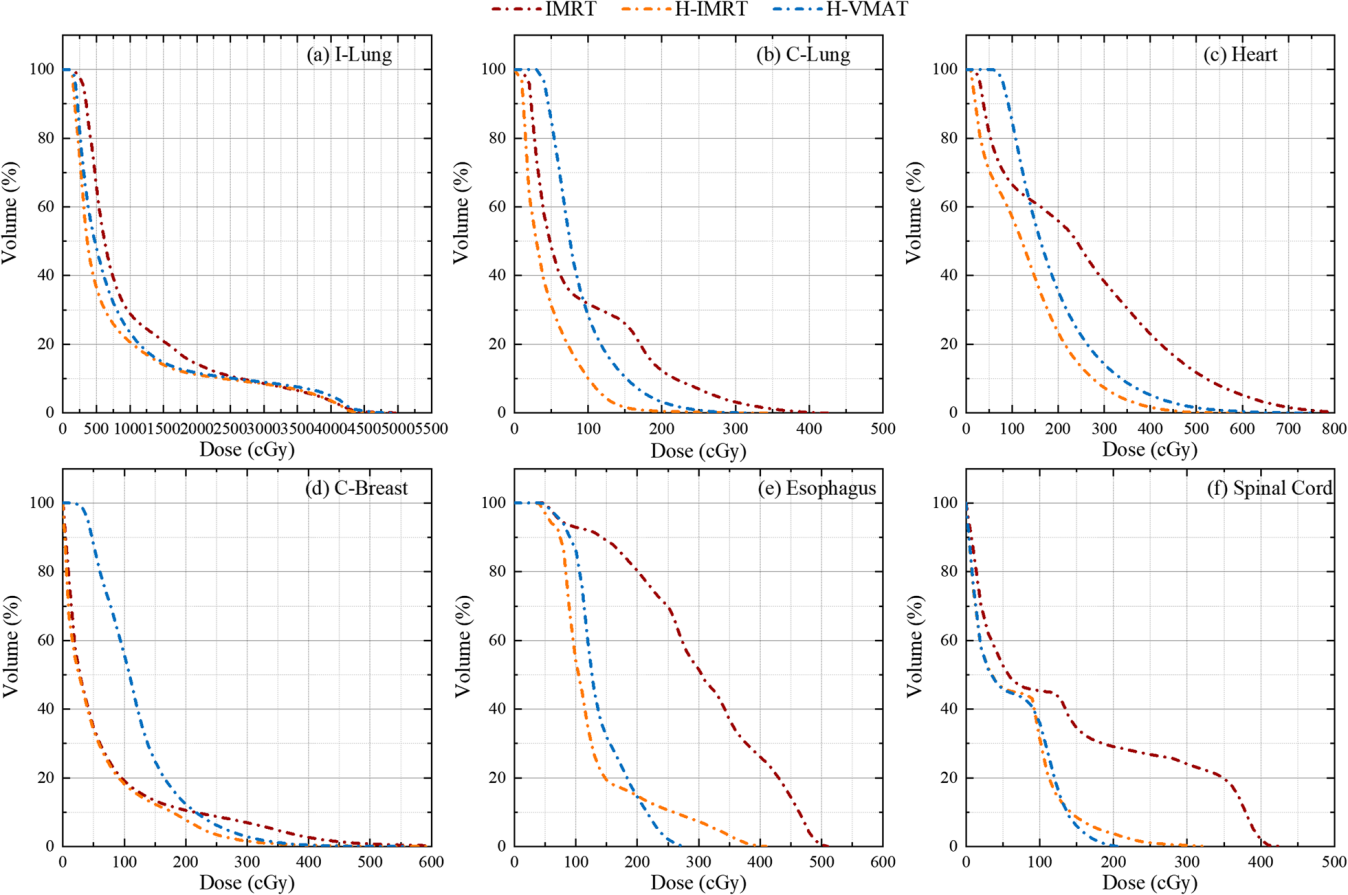
DVHs of OARs for the representative patient with IMRT (red line), Hybrid IMRT (orange line) and Hybrid VMAT (blue line). **a**–**f** are ipsilateral lung (I-Lung), contralateral lung (C-Lung), heart, contralateral breast (C-Breast), esophagus, and spinal cord, respectively

The DVHs of ipsilateral lung (I-Lung), contralateral lung (C-Lung), heart, contralateral breast (C-Breast), esophagus, and spinal cord of one representative case are displayed in Fig. 4a–f, respectively. The delivered doses to the OARs are listed in Table 4. Compared with the hybrid IMRT, V_5_ of ipsilateral lung, the Dmean of the esophagus with hybrid VMAT increased by 17.99% and149.27%, respectively (*p* = 0.046; 0.011), the V_10_ of ipsilateral lung with full IMRT increased 18.52% (*p* = 0.013), and the Dmean of the heart with hybrid VMAT dramatically increased by 4.67% compared with the hybrid VMAT (*p* = 0.033).

**Table 4:** Comparison of dosimetric parameters of OARs between IMRT, H-IMRT and H-VMAT

Structure	Parameters	IMRT	H-IMRT	H-VMAT	*p* value		
					a	b	c
I-Lung	V_5Gy_ (%)	32.78 ± 22.38	35.17 ± 6.05	41.50 ± 9.97	0.672	0.197	0.046
	V_10Gy_ (%)	24.19 ± 6.40	20.41 ± 3.75	19.44 ± 5.40	0.013	0.077	0.15
	V_20Gy_ (%)	14.84 ± 3.91	13.71 ± 3.14	13.47 ± 3.28	0.245	0.038	0.136
	V_30Gy_ (%)	10.38 ± 4.11	10.43 ± 2.79	10.37 ± 2.95	0.193	0.184	0.386
	D_mean_ (Gy)	11.71 ± 4.04	9.57 ± 4.58	10.36 ± 5.97	0.065	0.169	0.103
C-Lung	D_mean_ (Gy)	0.38 ± 0.42	0.34 ± 0.35	0.64 ± 0.43	0.842	0.16	0.068
Heart	V_5Gy_ (%)	5.05 ± 3.43	1.88 ± 2.89	2.78 ± 4.85	0.191	0.14	0.287
	V_10Gy_ (%)	0.35 ± 1.21	0.04 ± 0.12	0.07 ± 0.25	0.261	0.22	0.217
	D_mean_ (Gy)	1.63 ± 0.94	1.32 ± 0.65	1.71 ± 0.63	0.052	0.033	0.728
C-Breast	D_max_ (Gy)	12.59 ± 9.65	12.25 ± 13.09	11.81 ± 12.79	0.838	0.363	0.113
	D_mean_ (Gy)	0.83 ± 0.55	0.83 ± 0.50	0.99 ± 0.48	0.595	0.17	0.518
Esophagus	D_max_ (Gy)	3.38 ± 3.78	2.06 ± 1.66	3.78 ± 1.14	0.148	0.368	0.111
	D_mean_ (Gy)	1.10 ± 0.96	0.69 ± 0.47	1.72 ± 0.41	0.053	0.21	0.011
Spinal cord	D_max_ (Gy)	2.87 ± 3.30	2.13 ± 1.83	2.52 ± 0.76	0.362	0.542	0.376
	D_mean_ (Gy)	0.72 ± 1.32	0.58 ± 0.20	0.70 ± 0.24	0.552	0.485	0.184

a: IMRT versus H-IMRT; b: IMRT versus H-VMAT; c: H-IMRT versus H-VMAT

### SCR calculations

The EAR of the organs of contralateral breast, contralateral lung, ipsilateral lung, and esophagus with three treatment modalities are shown in Table 5. Compared with hybrid VMAT, the EAR to the contralateral breast with full IMRT and hybrid IMRT were decreased by 26.76% and 33.48%, respectively (*p* = 0.018; 0.031), and the EAR to the contralateral lung with full IMRT and hybrid IMRT were reduced by 48.05%, and 230.41%, respectively (*p* = 0.036; 0.000). In comparison with the hybrid IMRT, the EAR to the esophagus with full IMRT and hybrid VMAT increased 127.94% and 135.29%, respectively (*p* = 0.030; 0.014) and the EAR to the contralateral lung with full IMRT was increased 71.64% (*p* = 0.048).

**Table 5:** EAR comparison of OARs between IMRT, H-IMRT and H-VMAT

Structure	IMRT	H-IMRT	H-VMAT	*p* value		
				a	b	c
C-Breast	4.68 ± 3.39	4.25 ± 3.18	6.39 ± 3.71	0.205	0.018	0.031
I-Lung	109.80 ± 34.42	94.95 ± 39.35	115.53 ± 30.59	0.066	0.094	0.255
C-Lung	9.20 ± 8.06	5.36 ± 5.17	17.71 ± 6.02	0.048	0.036	0.000
Esophagus	1.55 ± 1.20	0.68 ± 0.54	1.60 ± 0.54	0.030	0.215	0.014

a: IMRT versus H-IMRT; b: IMRT versus H-VMAT; c: H-IMRT versus H-VMAT

## Discussion and conclusion

Since studies evaluating the hybrid IMRT and hybrid VMAT for early-stage breast cancer with SIB are rare, a comparison of the target dose coverage, OARs sparing, and SCR among full IMRT, hybrid IMRT, and hybrid VMAT for treating early-stage breast cancer with SIB is extremely relevant. This study aimed at estimate the three RT plans, and the expectation was to bring more clinical options to RT with SIB for early-stage right-sided breast cancer.

IMRT showed a significant advantage in target dose coverage, and surrounding OARs spring for left-sided breast cancer after breast-conserving surgery [8–10]. This could result in better tumor control rate and lower toxicity, and late effects compared with the conventional tangential pair treatment beams. However, IMRT had inherent geometrical uncertainties arising from setup error and target motion, which offset the merits of IMRT for breast cancer [10, 12, 29]. Combining two opposed tangential open beams and IMRT beams, the hybrid IMRT plan might solve the geometrical uncertainties of IMRT. Nakamura et al. [12] compared the plan quality and robustness of the dose distributions against setup and motion uncertainties among four RT plans. They confirmed that hybrid IMRT performed better robustness against the uncertainties than full IMRT, and it offered superior plan quality. Fogliata et al. [30] compared the dosimetric difference for the involved OARs among 3D-CRT plan with field in field technique, and two VMAT plans (VMAT_full and VMAT_tang, gantry rotation partial arc from about 295 to 173° without and with a sector of 0 MU, respectively) for breast cancer. They proved that full VMAT had an obvious weakness in radiating a higher mean dose to the nearby OARs compared with VMAT_tang.

Considering the excellent characteristics of hybrid plans and the lack of studies on hybrid VMAT plan, here, we eagerly studied the clinical dosimetric characteristics and SCR of full IMRT, hybrid IMRT, and hybrid VMAT, and we found that hybrid IMRT was superior to full IMRT and hybrid VMAT in target quality, and OARs sparing for early-stage right-sided breast cancer. Adopting the VMAT_tang (partial arcs with a sector of 0 MU) method from Fogliata et al.'s study, instead of two opposed tangential open beams plus a complete half arc in our study, the performance of hybrid VMAT in protecting peripheral OARs might be improved. However, different from irradiating the only target PTV as in Fogliata et al.'s study, the hybrid VMAT in our study delivered a boost dose to the tumor bed, and achieved better CI and HI for both the tumor bed and the PTV. Thus, the hybrid VMAT with a complete half arc beam might be reasonable in this study. However, the half arc beam delivered only 20% of the total dose by continuous rotation 180°, and the dose to the surrounding OARs inevitably increased. According to previous studies, it can be found that the plan quality for the IMRT and VMAT techniques depends a lot on the optimization process applied and the multiple beam's angles selected. In this study, in order to reduce the dose to the lung during the RT, the direction of 3D-CRT radiation field was still taken as the basis, and 10 degrees more was given to the outside and the gantry angle was range of about 200°, so as to increase the field regulation ability and achieve better uniformity for the target area.

As a tumor with a better therapeutic effect and longer life expectancy than most other tumors, the radiation-related risk is the most serious sequelae for breast cancer survivors, which has been confirmed by numerous epidemiological cohort studies [31]. The occurrence of secondary cancer is closely related to the tissues and organs themselves. Studies have shown that fatal secondary cancer mainly occurs in the stomach, lungs, and colon, and thyroid has a particularly low threshold of SCR (mean dose as low as 0.05 Gy in children and young adults) [31, 32]. In addition, the occurrence of secondary cancer depends on the radiation dose. Secondary cancer tends to occur in volumes receiving a total dose or near volumes receiving dose from 2 to 50 Gy radiation [31, 33]. Several studies demonstrated that SCR dramatically increased when receiving a dose reaching a certain range in the kidney (from 1 to 15 Gy), stomach and pancreas (from 1 to 45 Gy), and bladder and rectum (from 1 to 60 Gy) [30, 34]. In our study, seeking the least toxic radiation modality for breast cancer, we compared the SCR of three modalities for the contralateral breast, contralateral lung, ipsilateral lung, and esophagus.

Recently, Schneider proposed a calculation model, namely, the EAR model, which can be adopted for SCR calculation and evaluation utilizing DVH data from the RT plan and related radiobiological parameters [25, 28]. The EAR model has proved its feasibility to assess the SCR for patients with nasal natural killer T-cell lymphoma and breast cancer [28, 30]. Fogliata et al. [30] applied the EAR model to compare the SCR among 3D-CRT, VMAT_full, and VMAT_tang for breast cancer. And they confirmed that VMAT_tang had advantages in reducing RT toxicity for the ipsilateral organs compared with 3D-CRT with field in field technique when they delivered the same SCR to the contralateral organs.

In this study, we also adopted the EAR model to calculate the SCR for right-sided breast cancer, and our results demonstrated that the hybrid IMRT performed best in target quality, OARs spring, and SCR to peripheral OARs. However, if the half arc had a sector of 0 MU in hybrid VMAT, the performance of hybrid VMAT in SCR to adjacent OARs probably approached or achieved the effect of hybrid IMRT. The percentage of radiated dose and the effective dose delivery angle for the arc beam in the VMAT_tang in Fogliata's study and the hybrid VMAT in our study was quite different. This could translate into a differentiated radiation dose and SCR to the nearby healthy tissue. Of course, the results of the EAR model in predicting SCR depend on the accuracy of commercial TPS system modeling and related biological parameters.

In this study, EAR was used to quantify radiation-induced cancer. However, EAR is originally based on the risk calculations of extremely inhomogeneous dose distributions in the Hodgkin's cohort from the Japanese A-bomb survivors [26, 27] but not breast cancer cohort. It is also assumed that the total absolute risk in an organ is the volume weighted sum of the risks of the partial volumes which are irradiated homogeneously. In addition, uncertainties such as out of field low dose calculation as well as the effect of voxel size selection on dose calculation inevitably existed in commercial TPS.

Combining the results of previous studies with the results of this study, the following can be concluded: compared with 3D-CRT, IMRT and VMAT improved target uniformity in SIB treatment for early breast cancer, but increased second cancer risk. Barbara Dobler et al. [18, 35] found that compared to techniques with a limitation of short arcs or fields around the tangents for whole breast and SIB treatment, IMRT and VMAT are associated with a higher second cancer risk when exploiting a larger gantry angle range of around 200**°**. This conclusion is consistent with our research results. In addition, in our study, hybrid IMRT improved target uniformity and also had a lower second cancer compared with full IMRT and hybrid VMAT at the same gantry angel range.

Hybrid IMRT combined the advantages of 3D-CRT and IMRT in treating early-stage right-sided breast cancer. Hybrid IMRT was shown to have significant advantages in target dose coverage, OARs sparing, and SCR to nearby normal tissues. Hybrid IMRT is worthy of clinical application and promotion.

## Data Availability

Not applicable.

## Abbreviations

IMRT: Intensity-modulated radiotherapy
VMAT: Volumetric-modulated arc therapy
SCR: Second cancer risk
OAR: Organs at risk
SIB: Simultaneous integrated boost
EAR: Excess absolute risk
HI: Homogeneity index
RT: Radiotherapy
3D-CRT: 3-Dimensional conformal radiation therapy
MLC: Multi-leaf collimator
DR: Dose rate
CT: Computed tomography
TPS: Treatment planning system
CTV: Clinical target volume
PTV: Planning target volume
MUs: Monitor units
DVH: Dose-volume histogram
CI: Conformal index
